# Christmas in a "Wessex" County Hospital

**Published:** 1913-01-04

**Authors:** 


### CHRISTMAS IN A "WESSEX" COUNTY HOSPITAL.

Here in the Dorset County Hospital, in this picturesque building, with its Tudor style of architecture, in the town which Thomas Hardy has made famous as the "Casterbridge" of his novels, and which, by the way, can claim to be the native town of Sir Frederick Treves, Christmas was spent in a quiet, though by no means an unenjoyable manner. The day began by the singing of carols in the corridors at 6 a.m. by the maids, though long before then "stockings" hung out had been examined, more especially in the children's ward, where large muslin bags had been provided in order to hold the toys, dolls, scrap-books, and other articles dear to the heart of childhood. There was a service at 9.30 a.m. in the beautiful little chapel, followed by a celebration. The necessary morning work being then despatched, preparation was made for dinner, followed by dessert and a plentiful supply of bon-bons. Then followed the great treat for the men—tobacco, smoked in long churchwarden pipes, the gift of a thoughtful house surgeon. At intervals during the day music was heard in the wards. Each patient was allowed one visitor to tea and for the evening, and full justice was done to this sumptuous meal.

At 5.30 came the great event of the day. Father Christmas (impersonated by a member of the committee) arrived with his load. The venerable old gentleman first visited the children, who, wearing pale blue jackets and ribbons to tone with the colour scheme of their ward, gazed wonderingly at their visitor, scarcely heeding their gifts. On he went, next to the women's ward, where red and orange were the prevailing colours among the plants and flowers; and then to the men, in whose wards home-made poppies had been most successfully employed, with robins sitting about among the ivy and fairy lights. With each recipient he made a joke, no small feat, as they all called forth shrieks of laughter. His cart, resembling a vehicle about to enter a "Battle of Flowers" contest, followed him, and in it were found gifts for everyone, from the chairman and house surgeon down to the porter. The patients were delighted to find that in addition to at least one useful warm garment their parcels also contained something for mother, wife, or child. These gifts were all supplied by friends of the hospital, who responded liberally, with money or otherwise, to the appeal made to them. After wishing everyone a Happy New Year, the old gentleman departed amid hearty cheers, and the happy day was brought to a close by a short service in each ward by the Chaplain, and made gay with Christmas hymns. On Boxing Day gramophone selections were given by a friend, and impromptu concerts in the evening by the sisters and nurses, who for three days gave themselves up entirely to the patients. Without their ungrudging, cheerful service such a successful Christmas would have been impossible.

